# Hydrocalumite as well as the Formation of Scheelite Induced by Its Dissolution, Removing Aqueous Tungsten with Varying Concentrations

**DOI:** 10.3390/ijerph19148630

**Published:** 2022-07-15

**Authors:** Chen Yang, Qinghai Guo, Yaowu Cao, Georgii A. Chelnokov

**Affiliations:** 1 State Key Laboratory of Biogeology and Environmental Geology, School of Environmental Studies, China University of Geosciences, Wuhan 430074, China; yangchenjxnc@gmail.com (C.Y.); yaowu.cao0105@gmail.com (Y.C.); 2 Geological Institute, Russian Academy of Sciences, 119017 Moscow, Russia; geowater@mail.ru

**Keywords:** hydrocalumite, tungsten, anion exchange, precipitation, PHREEQC

## Abstract

As a toxic element, tungsten (W) in elevated concentrations, originating from human activities or geological sources, poses a severe threat to the environment. However, there has been a lack of robust remediation techniques focusing on aqueous tungsten contamination with varying initial concentrations, because only recently have the toxicity and the environmental threat of tungsten been fully realized. In this study, the removal of tungsten from an aqueous solution by hydrocalumite was investigated for the first time. Systematic removal experiments were carried out at designated contact time, temperature, and initial tungsten concentration. The results showed that hydrocalumite is capable of effectively removing tungsten under various conditions, especially at high initial tungsten concentrations, with the maximum uptake capacity being up to 1120.5 mg (tungsten)/g (hydrocalumite). The mechanisms of tungsten removal were studied based on the measured chemical compositions of the solution samples and their PHREEQC simulations as well as the solid sample characterization by XRD, SEM–EDX, and XPS. At low initial tungsten concentrations (below 1 mmol/L), anion exchange between the tungsten in solution and the Cl in the hydrocalumite interlayers played a critical role in tungsten removal. At high initial tungsten concentrations (higher than 5 mmol/L), the removal of W from the solution was solely caused by the precipitation of scheelite (CaWO_4_), facilitated by the substantial release of Ca^2+^ from hydrocalumite dissolution. At moderate tungsten concentrations (1–5 mmol/L), however, both mechanisms were responsible for the uptake of tungsten, with scheelite precipitation being more important. Hydrocalumite is promising for wide use in the treatment of high-tungsten natural waters or wastewaters.

## 1. Introduction

Tungsten is a transitional metal element in the periodic table and has been widely used in national defense construction and high-tech industries [1,2,3,4]. Since tungsten is non-toxic and has a weak ability to migrate in the environment when it exists in the form of a metal or an alloy, it was commonly regarded harmless to the environment and health [3]. However, a growing number of studies have shown that excessive tungsten has potential adverse effects on the environment and biological health [5,6,7]. For instance, a high dose of tungsten could lead to histopathological changes in the kidney and epididymis in rats [7]; high concentrations of tungsten in human urine or blood could cause spasm, stroke, or cardiovascular disease [8,9].

Excessive W in the environment comes from human activities or geological sources. Typical examples of anthropogenic tungsten include that in the pore water samples from several shooting ranges in the Massachusetts Military Reservation, with extremely high tungsten concentrations, ranging from 1 to 400 mg/L, due to five years of training with tungsten rounds [10]; in the groundwaters in the Yxsjöberg historical oxidic-sulfidic skarn tailings in Sweden, with tungsten concentrations as high as 922 mg/L [11]; and in the treated wastewaters from a science and technology park for semiconductor manufacture in Taiwan, with tungsten concentrations still up to 400 µg/L [12]. Representative of geogenic tungsten, the geothermal waters in the Semi magmatic hydrothermal system in Tibet have tungsten concentrations as high as 1103 μg/L [13]. Hence, the removal of tungsten from wastewaters, polluted waters, or naturally tungsten-rich waters, which may further contaminate other environmental media (such as surface waters or shallow groundwaters serving as a drinking water source), is of great importance. Correspondingly, development of cost-effective and efficient tungsten removal materials has become a hot spot in this field [14,15,16,17,18].

In recent years, the removal of tungsten from high-tungsten solutions has been realized mostly via adsorption methods. However, the tungsten uptake capacities of the reported adsorbents are generally not satisfactory and need to be improved. For example, multi-walled carbon nanotubes used to remove tungsten from an aqueous solution have a maximum tungsten adsorption capacity of only 17.6 mg/g [14]. Montmorillonite [16], pyrite [19], and kaolinite [20] are characterized by even lower tungsten adsorption capacities, of 4.9 mg/g, 13.2 mg/g, and 6.5 mg/g, respectively. Some hydrotalcite-like anion clays, owing to their large specific surface area, anion exchange capacity, and relatively weak interlamellar bonding, perform better in removing aqueous tungsten, such as nanocrystalline iowaite [21] and pyroaurite [22], with tungsten uptake capacities of 71.9 mg/g and 69.9 mg/g, respectively. Nevertheless, the above materials are only effective in removing tungsten from solutions with relatively low initial concentrations; that is, their tungsten uptake efficiencies would be significantly reduced if they were used to treat extremely high-tungsten waters, e.g., certain industrial wastewaters.

Superior to hydrotalcite-like anion clays, hydrocalumite-like anion clays generally feature a stronger capability of removing anionic pollutants with high concentrations, largely due to the situation that the layer constituting ions M^2+^/M^3+^ in hydrocalumite are Ca^2+^/Al^3+^ [23]. Hydrocalumite has been used to treat aqueous solutions containing various harmful anions, such as fluoride and arsenate [24,25], and successfully reduced the target components from high concentrations to a low concentration, primarily by precipitating them with Ca^2+^ substantially released from hydrocalumite via its dissolution. However, there have been so far no systematic experimental studies on the removal of tungsten from water using hydrocalumite. So, the aims of this study are to test the capability of hydrocalumite in tungsten uptake over a wide range of initial concentrations, to clarify the mechanisms involved in the removal of tungsten from water by hydrocalumite, and to evaluate the feasibility of employing hydrocalumite to remove tungsten from various high-tungsten waters.

## 2. Materials and Methods

### 2.1. Preparation and Characterization of Hydrocalumite

The chemicals used in this study were all of analytical grade or higher. All experiments were carried out under atmospheric conditions, with the solutions being prepared using ultrapure water. The sodium tungstate (Na_2_WO_4_·2H_2_O), sodium hydroxide (NaOH), anhydrous calcium chloride (CaCl_2_), and aluminum chloride (AlCl_3_·6H_2_O) used in this study were from Sinopharm Chemical Reagent Co., Ltd.

In this study, hydrocalumite was synthesized in the laboratory by the co-precipitation method [24]. A 250 mL mixture of ultrapure water and ethanol with the volume ratio of 2:3 (mixed solution 1) and a mixture of 0.66 mol/L CaCl_2_ and 0.33 mol/L AlCl_3_·6H_2_O (mixed solution 2) were prepared in advance. A 2 mol/L NaOH solution and the mixed solution 2 were added dropwise to the mixed solution 1. The NaOH solution was used to create an alkaline environment, ensuring that the pH of the solution was higher than 11. The whole dropwise addition process was carried out in a magnetic stirrer at 25 °C. After that, a white mud-like mixed solution was obtained and crystallized at 65 ± 3 °C for 24 h. The precipitation was centrifuged and washed repeatedly with ultrapure water until the conductivity was below 2.0 ms/cm. The solid products were dried in an oven at 50 ± 3 °C, ground in a mortar, and then passed through 60 target quasi-sieve for further use.

X-ray powder diffraction (XRD) was performed on a Bruker D8 advance powder X-ray Cu Ka radiation diffractometer for the synthetic hydrocalumite and the solid samples after they were reacted with tungsten-bearing solutions. The voltage of the process was 40 KV, the current was 30 mA, and the wavelength was 0.15406 nm. The X-ray diffraction patterns were presented in 2θ. In order to determine the compositions of the pristine and reacted solid samples, they were also subjected to a scanning electron microscope (SEM) analysis. The employed FEI Quanta 200 scanning electron microscope (SEM) was equipped with an energy dispersive X-ray (EDX) analysis capability. X-ray photoelectron spectroscopy (XPS) was performed on the synthetic hydrocalumite and parts of the reacted solid samples using a Thermo Scientific K-Alpha+ spectrometer adopting CAE scanning mode. The pass-energy of full spectrum scanning was 100 eV, with a step size of 1 eV, and that of narrow spectrum scanning was 30–50 eV, with a 0.05–0.1 eV step size. All spectra were charge corrected with C1s line at 284.8 eV.

### 2.2. Tungsten Removal Experiments

First, the reaction kinetics experiments were carried out. Sodium tungstate solutions with concentrations of 0.1, 1, and 20 mmol/L were prepared; placed in PET bottles; and reacted with 0.1 g added hydrocalumite powder in a QB-32B rotary incubator at 25 °C. After 5 min, 30 min, 1 h, 2 h, 5 h, 12 h, 18 h, and 24 h, the solution samples were collected and filtered, and pH, conductivity, and W concentration were measured. Then, isothermal experiments were conducted within the time frame determined in the reaction kinetics experiments. Specifically, sodium tungstate solutions with concentrations of 0.0001, 0.0005, 0.001, 0.005, 0.01, 0.02, 0.05, 0.1, 0.2, 0.5, 1, 2, 5, 10, 15, 20, 25, 30, and 40 mmol/L were reacted with hydrocalumite powder at 25 °C. After the reactions, the samples were filtered to determine their pH values, conductivities, and tungsten concentrations. The effects of temperature on tungsten removal were investigated at 45 °C, 65 °C, and 85 °C, with the results being compared with those at 25 °C. Batch experiments were also performed with 2 mM tungstate solutions over a wide pH range, of 2 to 12, which was adjusted by adding a small amount of dilute HCl or NaOH solutions. All of the above experiments were repeated twice, and the average experimental data were finally reported. Prior to the chemical analyses, all the samples were filtered through 0.22 µm membranes. The pH was measured using a Mettler Toledo pH meter, and the concentrations of the constituents in the solution samples were measured by ICP–MS and IC, the former of which has been widely used for analysis of metal ions in various samples in view of its high sensitivity, low detection limits, and simultaneous multi-element analysis capability [26,27,28,29,30,31,32].

### 2.3. PHREEQC Modeling

PHREEQC is a computer software package for calculating a wide range of hydrogeochemical reactions. In this study, it was used to simulate the removal of tungsten from an aqueous solution by hydrocalumite. Specifically, the pH values as well as the concentrations of tungsten and other ions in the solutions at equilibrium were estimated by the PHREEQC code. The amounts of hydrocalumite and scheelite that may be dissolved in or precipitated from the solutions were calculated as well. Due to the multi-factorial nature of the experiments, the simulative results are not completely in accordance with the experimental data. However, to simulate the removal of tungsten from an aqueous solution by hydrocalumite in advance using PHREEQC is of great significance for the design of the experiments. The comparison between the simulative results and the experimental data is also helpful for a better understanding of the mechanisms involved in tungsten removal by hydrocalumite.

## 3. Results and Discussion

### 3.1. Characterization of Synthetic Hydrocalumite

As shown in Figure S1a, the XRD pattern of the hydrocalumite synthesized via co-precipitation has a smooth baseline and matches well with the standard PDF card of hydrocalumite. The diffraction peaks are all characteristic of typical hydrocalumite crystals and are essentially free of spurious peaks. Notably, the diffraction peak on the (002) crystal plane corresponding to the low 2θ region is sharp, indicating that the synthetic hydrocalumite is pure and has crystallized into a complete monoclinic symmetrical lamellar structure with a single crystalline phase and a high degree of interlayer regularity. The spacing of the (002) crystal plane is 7.80 Å.

The synthetic hydrocalumite crystals were visualized via SEM (Figure S1b), showing a lamellar structure, with the better developed ones being regular ortho-hexagonal plate-like. EDX analyses reveal that the Ca:Al molar ratio of the hydrocalumite is 1.95:1, close to the theoretical value (2:1). In comparison, the Cl-to-Al-molar ratio is equal to 0.87, below the theoretical value (1), indicating that some hydroxyl ions may have entered the intercalation regions during the synthesis of hydrocalumite in a highly alkaline environment.

### 3.2. Kinetic and Isothermal Experimental Studies of Tungsten Removal

The kinetic processes of tungsten removal at 25 °C with initial tungsten concentrations of 0.1, 1, and 20 mmol/L are shown in Figure S2. The kinetic curves at the three different initial concentrations followed a similar trend: the W concentrations in the solutions all decreased rapidly within the first 2 h and then leveled off, with no significant changes in the aqueous tungsten concentrations after 5 h. The data were fitted to a reaction kinetic model. As shown in Figure S3, the kinetic data for tungsten removal by hydrocalumite before the reaction equilibrium matched well with the regular second-order dynamics model.

The results of the isothermal experimental study are shown in Figure S4, demonstrating that the amount of tungsten removed by hydrocalumite changed linearly with the initial tungsten concentration over the range of 0.0001–15 mmol/L. At an initial concentration of 15 mmol/L, the removal amount reached the highest level, after which the removal amount remained nearly constant regardless of the initial aqueous tungsten concentration. It is interesting that the tungsten removal percent gradually increased at relatively low initial tungsten concentrations, reached the top at an initial concentration of 5 mmol/L, and then decreased with further increasing initial tungsten concentrations (Table S1).

**Figure 1:**
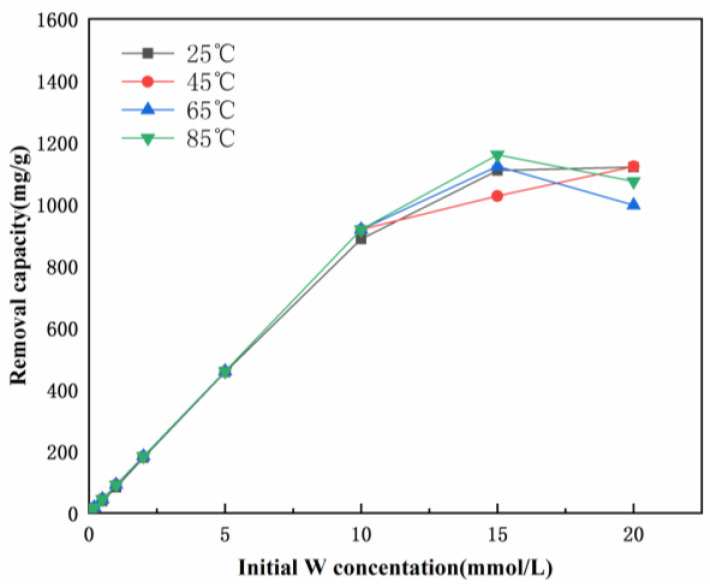
Tungsten uptake by hydrocalumite at 25, 45, 65, and 85 °C.

Moreover, the effects of reaction temperature on tungsten removal by hydrocalumite are shown in Figure 1. At temperatures ranging from 25 to 85 °C, the tungsten uptake capacities of hydrocalumite are almost the same. Therefore, temperature has little influence on the tungsten removal efficiency of hydrocalumite.

### 3.3. Effects of pH on Tungstate Removal

**Figure 2:**
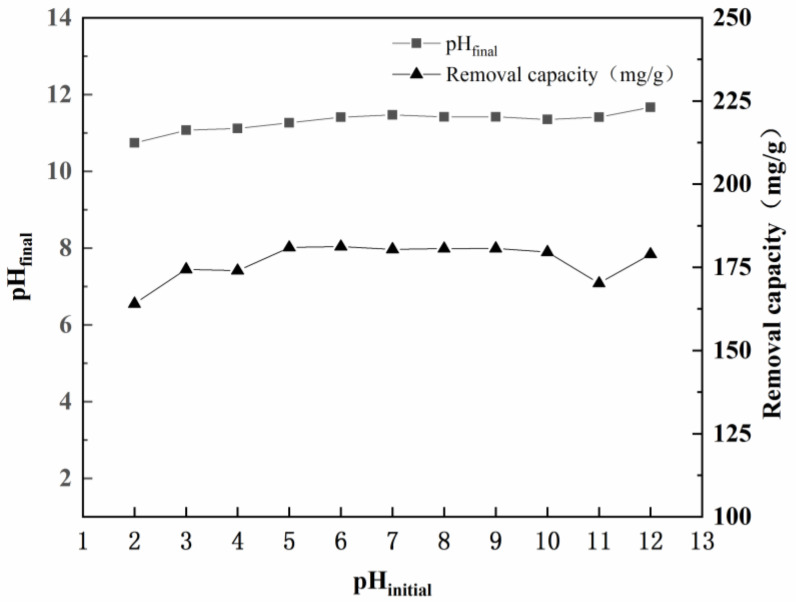
Effects of initial solution pH on tungsten removal by hydrocalumite.

The solution pH is an important parameter that affects the efficiency of practical water treatment cases. In this experimental study, tungstate solutions with different initial pH values, ranging from 2 to 12, were prepared to investigate the effects of solution pH on tungsten removal by hydrocalumite (Figure 2). As shown in Table S2, the final pH values of the solutions after reacting with hydrocalumite varied from 10.74 to 11.67, due to the strong pH buffering effect of hydrocalumite. Correspondingly, the initial pH of the tungsten-bearing solution had little effect on the tungsten uptake capacity of hydrocalumite. Only at an extremely acidic initial pH (pH = 2) could a small decrease in the uptake capacity, from 180 mg/g (the maximum) to 164 mg/g, be observed.

### 3.4. Characterization of Solid Samples Reacted with Solution Tungsten

#### 3.4.1. X-ray Diffraction (XRD) and Scanning Electron Microscopy (SEM)

**Figure 3:**
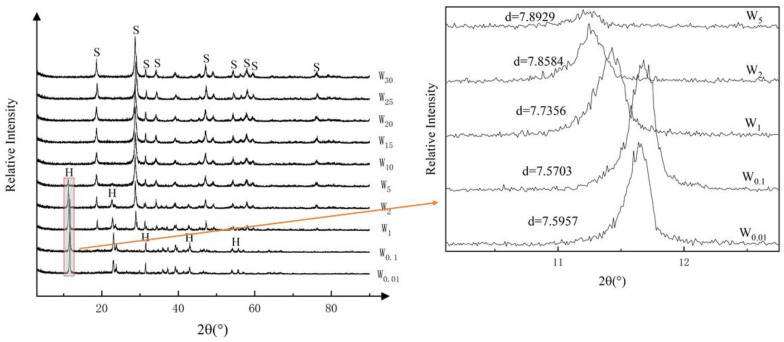
XRD patterns of hydrocalumite after reaction with tungsten. H: hydrocalumite; S: scheelite. The sample numbers beside the curves indicate the initial tungsten concentration in solution (in mmol/L).

The XRD patterns of the solid samples recovered from the tungsten removal experiments are shown in Figure 3. The primary peak of hydrocalumite became progressively weaker at initial tungsten concentrations from 0.1 to 5 mmol/L; beyond 5 mmol/L, the peak of hydrocalumite was completely absent. Correspondingly, the peak of scheelite became stronger and stronger on increasing the initial tungsten concentrations from 1 mmol/L to 30 mmol/L.

**Figure 4:**
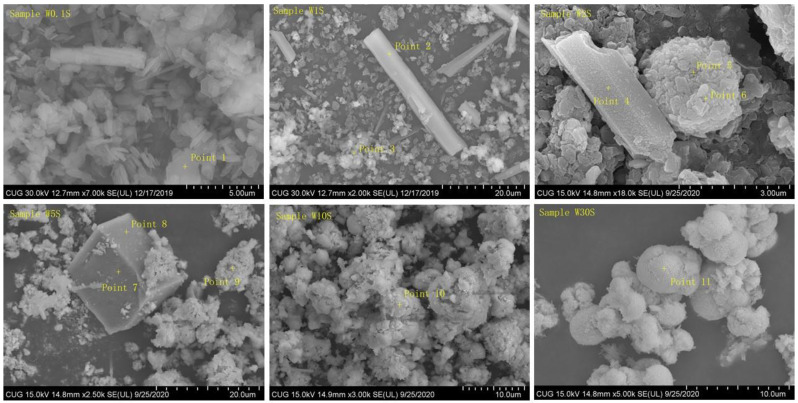
SEM images of hydrocalumite samples after reaction with tungstate solution. Point 1–11 are the locations of the dots on the sample during EDX analysis.

Several typical reacted solid materials were also subjected to SEM–EDX analyses, with the results being shown in Figure 4. When the initial solution tungsten concentration was low (0.1 mmol/L), hexagonal crystals and a few columnar crystals were observed under SEM. The EDX analysis (Table S3) indicated that the hexagonal crystals were still hydrocalumite, while the columnar crystals may be a calcium carbonate-like mineral. The absence of peaks corresponding to any calcium carbonate minerals in the XRD patterns was possibly due to their low contents. In contrast, the solid sample recovered from the experiment with an initial tungsten concentration of 2 mmol/L did not show any intact hexagonal crystals; instead, irregular clusters of crystals and columnar crystals were ubiquitous. Still, the columnar crystals were likely a calcium carbonate-like mineral, while the irregular clusters next to them were mainly composed of O, Ca, and tungsten, with elemental ratios almost identical to those of CaWO_4_, revealing that they were less crystalline scheelite. When the initial tungsten concentration was increased to 30 mmol/L, well-crystallized spherical clusters were observed, which were confirmed by XRD and EDX as scheelite crystals.

#### 3.4.2. X-ray Photoelectron Spectroscopy (XPS)

**Figure 5:**
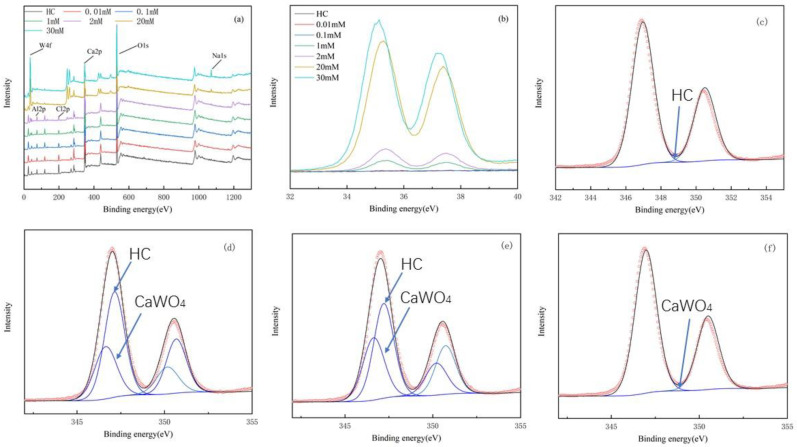
XPS analysis of hydrocalumite before and after its reaction with aqueous tungstate. Full range (**a**); W4f spectra of solids after tungsten removal at different initial tungsten concentrations (**b**); Ca2p spectra of hydrocalumite (**c**); Ca2p spectra of solids after tungsten removal at initial tungsten concentrations of 1 mmol/L (**d**), 2 mmol/L, (**e**) and 30 mmol/L (**f**). (**c**–**f**): ○ Original data; – Fitting results.

The full XPS spectra of the reacted solid samples (Figure 5a) showed that the peaks of Al2p and Cl2p gradually weakened with increasing initial solution tungsten concentrations. The W4f spectra (Figure 5b), in contrast, demonstrated that although there were no characteristic peaks of tungsten in the unreacted sample and the samples reacted with the solutions with low initial tungsten concentrations (such as 0.01 and 0.1 mmol/L), clear double peaks with binding energies ranging from 33 to 39 eV appeared as the initial tungsten concentration increased. In addition, the intensity of these peaks increased with increasing initial tungsten concentrations, implying that the reacted solid samples did form a chemical bond with the tungsten in the solutions.

Further peak splitting fits were made to the Ca2p spectra of the pristine hydrocalumite and the reacted solid samples. The XPS binding energy for the 3/2p Ca2p orbital in CaWO_4_ (around 346.5 eV) and those for the 3/2p and 1/2p Ca2p orbitals in chloride intercalated hydrocalumite (347 and 350.6 eV) are from the NIST database (http://srdata.nist.gov/xps/Default.aspx, accessed on 7 March 2021) and [33], respectively. As shown in Figure 5c–f and Table S4, the percentage of hydrocalumite-containing Ca^2+^ in the pristine hydrocalumite sample was 100%, as expected. With the increase in the initial solution W concentrations, the percentage of hydrocalumite-containing Ca^2+^ in the reacted samples gradually decreased. When the initial tungsten concentrations increased to 30 mmol/L, all the Ca in the solid samples became CaWO_4_-containing Ca^2+^, indicating once again that the hydrocalumite originally added to the reaction system was used up and scheelite had substantially formed. These results were consistent with the XRD and SEM characterizations of the solid samples.

### 3.5. Chemical Compositions of Tungsten-Bearing Solutions after Reaction with Hydrocalumite and Their PHREEQC Simulations

To better understand the experimental results, the pH values as well as the concentrations of Ca, Al, Cl, Na, and W and the alkalinity of the solution samples after reaction with hydrocalumite were measured, based on which the activities of Ca^2+^, Cl^−^, ${\mathrm{Al(OH)}}_{4}^{-}$, H^+^, OH^−^, and ${\mathrm{WO}}_{4}^{2-}$ were calculated using PHREEQC. Then the ionic activity products (IAPs) of the relevant minerals that probably dissolved in or precipitated from the solutions, such as hydrocalumite, gibbsite (Al(OH)_3_), bayerite (Al(OH)_3_), portlandite (Ca(OH)_2_), and scheelite (CaWO_4_), were calculated and compared with their solubility product constants (KSPs). The solubility product constants (KSPs) for these minerals are shown in Table S5.

**Figure 6:**
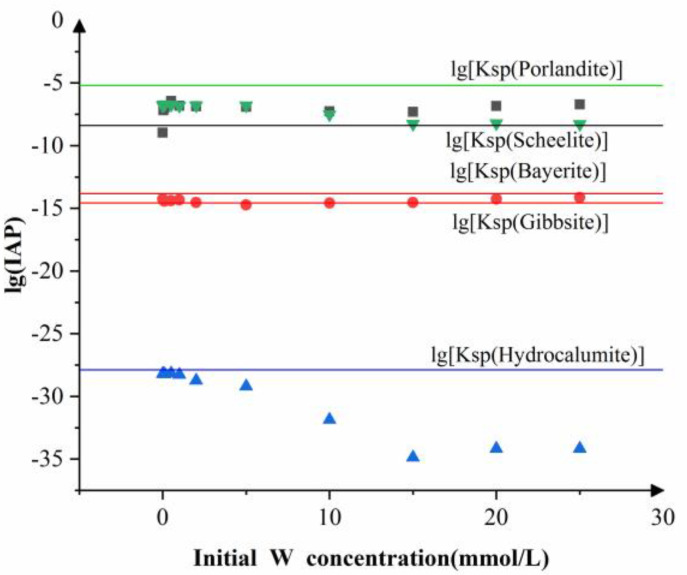
Solubility product constants for minerals in the reaction system versus the activity products of the corresponding component ions. Symbols: ▲ hydrocalumite; ● gibbsite or bayerite; ■ scheelite; ▼ portlandite.

As shown in Figure 6, the lg(IAP) values of the solutions were much lower than the lg(KSP) value of portlandite and therefore it was unlikely to precipitate. Similarly, bayerite appeared to have little chance of formation at any initial tungsten concentrations. In contrast, the calculated lg(IAP) values for gibbsite were close to the corresponding lg(KSP) line, indicating that it tended to precipitate out of solution during the experiments. More complicatedly, the solution samples were not saturated with respect to scheelite at low initial tungsten concentrations but became supersaturated at higher concentrations, implying that the formation of scheelite at higher initial tungsten concentrations was responsible for tungsten removal. When the initial tungsten concentrations were below 5 mmol/L, the lg(IAP) and lg(KSP) values of hydrocalumite were almost the same, so not all the hydrocalumite added to the solution was dissolved at these low initial tungsten concentrations. However, as the initial tungsten concentrations further increased, the lg(IAP) of the solutions with respect to hydrocalumite decreased to values well below its lg(KSP) value, indicating that hydrocalumite could be used up once the initial tungsten concentrations were high enough.

**Figure 7:**
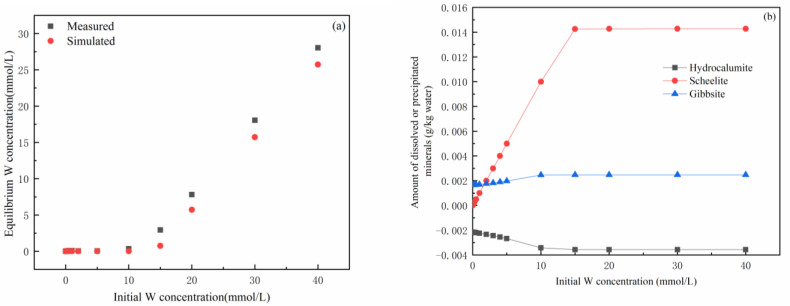
PHREEQC simulations. Simulated tungsten concentrations at equilibrium and their comparison with the measured values (**a**). PHREEQC simulation of the amount of minerals that may dissolve or precipitate in the reaction (**b**).

The above information demonstrated that the dissolution of hydrocalumite and subsequent precipitation of scheelite should be the most important mechanism related to the removal of tungsten from the solutions. Therefore, we employed PHREEQC, which is capable of simulating dissolution–precipitation processes, to calculate the tungsten concentrations in the reacted solutions and the dissolved or precipitated amounts of the minerals involved in this experimental study. As shown in Figure 7a, the measured tungsten concentrations in the solutions at equilibrium are close to the simulated concentrations, especially when the initial solution tungsten concentrations are relatively low. Likewise, the predictions for the dissolution of hydrocalumite as well as the precipitation of gibbsite and scheelite by PHREEQC matched well with the actual observations (Figure 7b). At low initial W concentrations, the hydrocalumite could not be completely reacted and less scheelite was produced. At higher initial tungsten concentrations, however, more Ca^2+^ released from the dissolution of hydrocalumite was consumed to form scheelite; accordingly, more tungsten was removed from the solutions. In addition, the simulation results show that a small amount of gibbsite is produced at different initial W concentrations due to the release of a considerable amount of Al available for gibbsite formation from hydrous calcium alumina during CaWO4 formation. In other words, the PHREEQC simulations confirmed the vital role of scheelite precipitation in the W removal process.

### 3.6. Tungsten Removal Mechanisms

As indicated by the chemical compositions of the solution samples; the PHREEQC simulations; and the solid sample characterization by XRD, SEM–EDX, and XPS, the dissolution of hydrocalumite and the precipitation of scheelite were the predominant mechanisms of tungsten removal from solution, which can be expressed via the following reactions.

$${\mathrm{Ca}}_{4}{\mathrm{Al}}_{2}{\left(\mathrm{OH}\right)}_{12}{\mathrm{Cl}}_{2}\cdot 6H_{2}O\;=\;4{\mathrm{Ca}}^{2+}\;+\;2\mathrm{Al}{\left(\mathrm{OH}\right)}_{4}^{-}\;+\;2{\mathrm{Cl}}^{-}\;+\;4{\mathrm{OH}}^{-}\;+\;6H_{2}O$$

$${\mathrm{Ca}}^{2+}{+\mathrm{WO}}_{4}^{2-}{=\mathrm{CaWO}}_{4}↓$$

Nevertheless, the above dissolution–precipitation reactions should not be the sole process controlling the concentrations of tungsten in the solutions. At low initial tungsten concentrations, there was no evidence for the occurrence of scheelite precipitation, but the tungsten in the solutions was still removed somehow (though not significantly effective). For the experiments with low initial tungsten concentrations (from W0.01S to W5S), the hydrocalumite originally added was at least partially present until the end of the experiments and, therefore, the removal of tungsten could be achieved to various degrees by its exchange with the chloride ions in the hydrocalumite interlayers. Indeed, the EDX elemental analysis showed the presence of tungsten in the hexagonal hydrocalumite crystals. In fact, only at initial tungsten concentrations above 5 mmol/L could all the added hydrocalumite be consumed and the formation of scheelite became the sole process removing tungsten from the solution.

## 4. Conclusions

In this study, hydrocalumite was synthesized by the coprecipitation method and used to treat aqueous solutions with various tungsten concentrations. The uptake capacity of tungsten by hydrocalumite was superiorly high (with the highest being 1120 mg (tungsten)/g (hydrocalumite)), several times higher than those of other materials commonly used for W removal. The removal of tungsten by hydrocalumite was in line with the regular second-order kinetics model and was little affected by variation in reaction temperature. The mechanisms of tungsten removal involved in this study could be anion exchange, dissolution–precipitation, or a combination of them. At low initial tungsten concentrations (below 1 mmol/L), anion exchange between solution tungsten and interlayer Cl was the predominant process removing tungsten, provided that scheelite was not produced in the reaction system; at high initial tungsten concentrations (higher than 5 mmol/L), scheelite precipitation was the sole mechanism for tungsten uptake because the added hydrocalumite was completely consumed. Both mechanisms, however, made contributions to the removal of tungsten at moderate initial tungsten concentrations (1–5 mmol/L) in view that the originally added hydrocalumite and precipitated scheelite coexisted in the reaction system.

## Data Availability

All data included in this study are available upon request by contacting the corresponding author.

## Supplementary Materials

The following supporting information can be downloaded at https://www.mdpi.com/article/10.3390/ijerph19148630/s1: Figure S1: Characterization of hydrocalumite synthesized by coprecipitation method; Figure S2: Variation of solution tungsten concentrations with time at initial concentrations of 0.1 (a), 1 (b) and 20 (c) mmol/L; Figure S3: Reaction kinetics model fitting diagram; Figure S4: Experimental results of tungsten removal by hydrocalumite at 25 °C; Table S1: Experimental results of tungsten removal by hydrocalumite at 25 °C; Table S2: Solubility product constants (KSP) for the mineral phases involved in the PHREEQC modeling; Table S3: Results of EDX analysis of elemental composition for solid samples recovered from tungsten removal experiments; Table S4: Fitting parameters for Ca 2p3/2; Table S5: Solubility product constants (KSP) for the mineral phases involved in the PHREEQC modeling. Refs. [34,35] are cited in the supplementary files.
