# Supplementary material for: Hydrocalumite as well as the Formation of Scheelite Induced by Its Dissolution, Removing Aqueous Tungsten with Varying Concentrations
Source: Int J Environ Res Public Health. 2022 Jul 15;19(14):8630. doi: 10.3390/ijerph19148630 (PMC9323804; doi:10.3390/ijerph19148630)
Supplement: Supplementary file 1 [file ijerph-19-08630-s001.zip › Table S1.pdf]

Table S1. Experimental results of tungsten removal by hydrocalumite at 25℃.

Initial W concentration (mmol/L)	Equilibrium W concentration (mmol/L)	Adsorption capacity(mg/g)	W removal percent (%)	pH_{final}
0.0001	0.00006	0.0035	40.0	11.95
0.0005	0.0002	0.029	60.0	11.94
0.001	0.0004	0.057	60.0	12.02
0.005	0.0021	0.265	58.0	12.00
0.01	0.0042	0.533	58.0	11.98
0.02	0.0077	1.135	61.5	11.96
0.05	0.0136	3.347	72.8	11.95
0.1	0.0225	7.127	77.5	11.96
0.2	0.0367	15.01	81.7	11.94
0.5	0.074	39.15	85.2	11.98
1	0.09	83.64	91.0	11.94
2	0.05	179.5	97.5	12.00
5	0.04	456.1	99.2	12.08
10	0.34	888.0	96.6	12.20
15	2.93	1109.9	80.5	12.31
20	7.81	1120.5	61.0	12.32
25	13.03	1100.6	47.9	12.35
30	18.05	1098.0	39.8	12.40
40	28.04	1099.1	29.9	12.43
