# Supplementary material for: Hydrocalumite as well as the Formation of Scheelite Induced by Its Dissolution, Removing Aqueous Tungsten with Varying Concentrations
Source: Int J Environ Res Public Health. 2022 Jul 15;19(14):8630. doi: 10.3390/ijerph19148630 (PMC9323804; doi:10.3390/ijerph19148630)
Supplement: Supplementary file 1 [file ijerph-19-08630-s001.zip › Table S2.pdf]

Table S2. Effect of initial pH of the solution on the effect of tungsten removal by hydrocalumite (solid-liquid ratio = 2 g/L).

number	$pH_{initial}$	Initial W concentration （mmol/L）	Equilibrium W concentration （mg/L）	pH_{final}	Adsorption capacity （mg/g）	W removal percent （%）
pH2	2.05	2	39.57	10.74	164.06	89.24
pH3	2.97	2	18.92	11.07	174.38	94.85
pH4	4.06	2	19.70	11.12	173.99	94.64
pH5	5.07	2	5.80	11.26	180.94	98.42
pH6	6.05	2	5.26	11.41	181.21	98.57
pH7	7.09	2	6.86	11.47	180.41	98.13
pH8	8.05	2	6.45	11.42	180.61	98.24
pH9	9.01	2	6.39	11.42	180.65	98.26
pH10	9.93	2	8.53	11.35	179.58	97.68
pH11	10.99	2	7.28	11.41	170.20	92.58
pH12	11.93	2	9.79	11.67	178.94	97.34
