# Supplementary material for: Hydrocalumite as well as the Formation of Scheelite Induced by Its Dissolution, Removing Aqueous Tungsten with Varying Concentrations
Source: Int J Environ Res Public Health. 2022 Jul 15;19(14):8630. doi: 10.3390/ijerph19148630 (PMC9323804; doi:10.3390/ijerph19148630)
Supplement: Supplementary file 1 [file ijerph-19-08630-s001.zip › Table S3.pdf]

Table S3. Results of EDX analysis of elemental composition for solid samples recovered from tungsten removal experiments.

Sample number	Point number	Atomic percent(%)						
		Ca	Al	O	W	Cl	Na	C
W0.1S	1	32.3	6.8	45.6	0	2.2	0	12.3
W1S	2	27.4	0.86	49.5	0.1	0.3	0	21.6
W1S	3	10.6	42.5	10.7	1.4	2.0	0	32.3
W2S	4	14.4	2.3	53.9	0.3	0.6	0	27.3
W2S	5	10.5	1.6	57.8	8.0	1.5	0	14.5
W2S	6	10.1	4	57.9	6.2	2.2	0	15.2
W5S	7	39.3	0	46.0	0	0	0	12.9
W5S	8	13.4	0.2	60.0	0.2	0	0	25.6
W5S	9	11.7	3.0	57.5	9.4	0.4	0.9	13.6
W10S	10	13.4	1.9	56.1	11.4	0	1.3	10.9
W30S	11	13.9	1	46.5	12.5	1.7	0	15.2

(The rationale for the sample labels appearing in the tables, figures and text is as follows: The letter W refers to tungsten. The number in the middle indicates the initial concentration of sodium tungstate in the solution. The letter S represents a solid sample. For example, the sample number W0.1S refers to the solid material after the reaction of hydrocalumite with sodium tungstate solution with an initial concentration of 0.1 mol/L .)
