# Supplementary material for: Hydrocalumite as well as the Formation of Scheelite Induced by Its Dissolution, Removing Aqueous Tungsten with Varying Concentrations
Source: Int J Environ Res Public Health. 2022 Jul 15;19(14):8630. doi: 10.3390/ijerph19148630 (PMC9323804; doi:10.3390/ijerph19148630)
Supplement: Supplementary file 1 [file ijerph-19-08630-s001.zip › Table S4.pdf]

Table S4. Fitting parameters for Ca 2p3/2.

sample	HC				$CaWO_4$			
	BE		FWHM	%	BE		FWHM	%
	Ca2p 1/2	Ca2p 3/2			Ca2p 1/2	Ca2p 3/2		
HC	350.5	346.97	1.51	100				
W1S	350.67	347.15	1.41	66.7	350.17	346.64	1.54	33.3
W2S	350.73	347.21	1.41	60.2	350.2	346.64	1.5	39.8
W30S					350.18	346.61	1.59	100

(HC: pristine hydrocalumite)
