# Supplementary material for: Hydrocalumite as well as the Formation of Scheelite Induced by Its Dissolution, Removing Aqueous Tungsten with Varying Concentrations
Source: Int J Environ Res Public Health. 2022 Jul 15;19(14):8630. doi: 10.3390/ijerph19148630 (PMC9323804; doi:10.3390/ijerph19148630)
Supplement: Supplementary file 1 [file ijerph-19-08630-s001.zip › Table S5.pdf]

Table S5. Solubility product constants (KSP) for the mineral phases involved in the PHREEQC modeling.

Mineral phase	Formula	Dissolution reaction	log KSP	Source
Hydrocalumite	$Ca_4Al_2(OH)_{12}Cl_2{\cdot}6H_2O$	$Ca_4Al_2(OH)_{12}Cl_2{\cdot}6H_2O = 4Ca^{2+} + 2Al(OH)_4^- + 2Cl^- + 4OH^- + 6H_2O$	-27.89	[34]
Portlandite	$Ca(OH)_2$	$Ca(OH)_2 = Ca^{2+} + 2OH^-$	-5.20	WATEQ4F database
Gibbsite	$Al(OH)_3$	$Al(OH)_3 + H_2O = Al(OH)_4^- + H^+$	-14.56	WATEQ4F database
Bayerite	$Al(OH)_3$	$Al(OH)_3 + H_2O = Al(OH)_4^- + H^+$	-13.82	[35]
Scheelite	$CaWO_4$	$CaWO_4 = Ca^{2+} + WO_4^{2-}$	-8.38	SOLTHERM-XPT database

References

34. Bothe, J. V.; Brown, P. W. PhreeqC modeling of Friedel's salt equilibria at 23 +/- 1 degrees C. *CEMENT CONCRETE RES.* **2004**, 34, (6), 1057-1063. https://doi.org/10.1016/j.cemconres.2003.11.016.

35. Wagh, A. S., Chemically Bonded Phosphate Ceramics: Twenty-First Century Materials with Diverse Applications. *Elsevier Science* **2004**, 283.
