# Supplementary figures and images for: Hydrocalumite as well as the Formation of Scheelite Induced by Its Dissolution, Removing Aqueous Tungsten with Varying Concentrations
Source: Int J Environ Res Public Health. 2022 Jul 15;19(14):8630. doi: 10.3390/ijerph19148630 (PMC9323804; doi:10.3390/ijerph19148630)

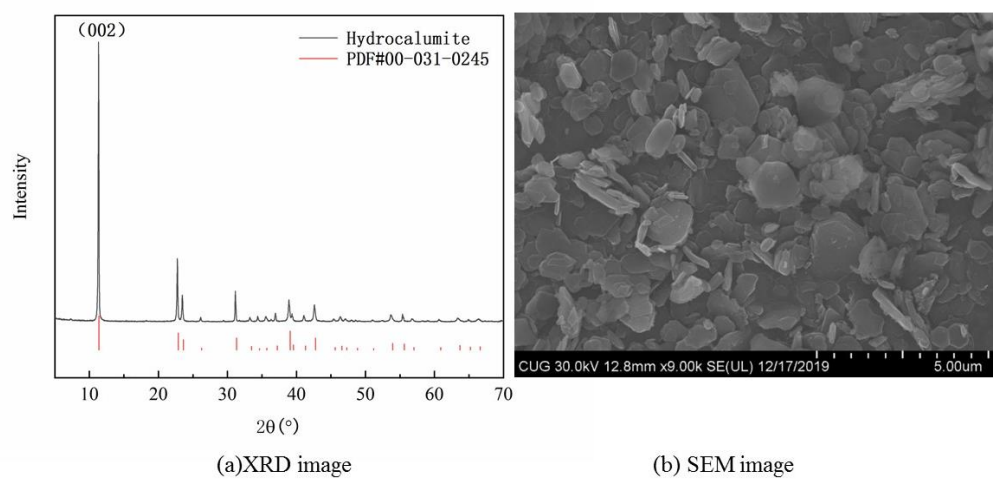

**Figure S1.** Characterization of hydrocalumite synthesized by coprecipitation method.

Supplement: Supplementary file 1 [file ijerph-19-08630-s001.zip › Figure S1.pdf]

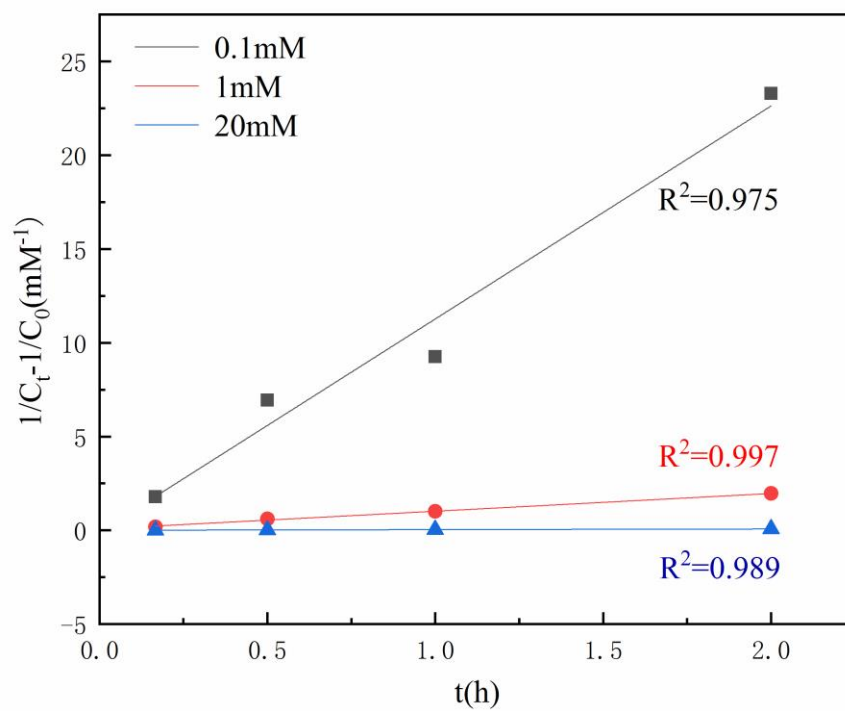

**Figure S3.** Reaction kinetics model fitting diagram.

Supplement: Supplementary file 1 [file ijerph-19-08630-s001.zip › Figure S3.pdf]

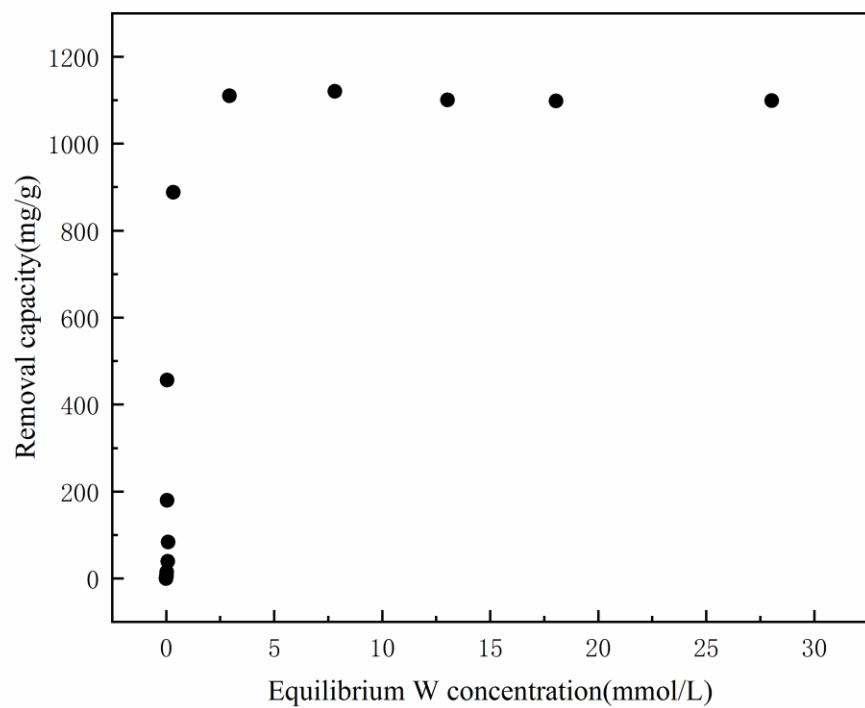

**Figure S4.** Experimental results of tungsten removal by hydrocalumite at 25°C.

Supplement: Supplementary file 1 [file ijerph-19-08630-s001.zip › Figure S4.pdf]
